# Poor self-care practices and contributing factors among adults with type 2 diabetes in Adama, Ethiopia

**DOI:** 10.1038/s41598-024-63524-8

**Published:** 2024-06-13

**Authors:** Nardos Tilahun Bekele, Ephrem Mannekulih Habtewold, Haji Aman Deybasso, Yohannes Mekuria Negussie

**Affiliations:** 1 Department of Public Health, Adama Hospital Medical College, Adama, Ethiopia; 2 Department of Medicine, Adama General Hospital and Medical College, Adama, Ethiopia

**Keywords:** Self-care practices, Type 2 diabetes, Associated factors, Adama, Ethiopia, Endocrinology, Risk factors

## Abstract

Diabetes mellitus (DM) is a prominent global health challenge, characterized by a rising prevalence and substantial morbidity and mortality, especially evident in developing nations. Although DM can be managed with self-care practices despite its complexity and chronic nature, the persistence of poor self-care exacerbates the disease burden. There is a dearth of evidence on the level of poor self-care practices and contributing factors among patients with DM in the study area. Thus, this study assessed the proportion of poor self-care practices and contributing factors among adults with type 2 DM in Adama, Ethiopia. An institution-based cross-sectional study was conducted among 404 patients. Self-care practice was assessed by the summary of diabetes self-care activities questionnaires. Binary logistic regression was used to identify factors associated with poor self-care practices. An adjusted odds ratio with a 95% confidence interval was used to assess the strength of associations. The statistical significance was declared for a *p*-value < 0.05. The proportion of poor self-care practices was 54% [95% CI 49.1, 58.6]. Being divorced (AOR = 3.5; 95% CI 1.0, 12.2), having a lower level of knowledge (AOR = 1.70; 95% CI 1.0, 2.8), being on insulin (AOR = 6.3; 95% CI 1.9, 20.6), taking oral medication (AOR = 8.6; 95% CI 3.0, 24.5), being unaware of fasting blood sugar (AOR = 2.9; 95% CI 1.6, 5.2), not a member of a diabetic association (AOR = 3.6; 95% CI 1.7, 7.5), a lack of social support (AOR = 2.9; 95% CI 1.7, 4.9), and having a poor perceived benefit of self-care practices (AOR = 1.84; 95% CI 1.0, 3.2) were associated with poor self-care practices. Overall, this finding demonstrated that a significant percentage of participants (54%) had poor self-care practices. Being divorced, having a low level of knowledge about diabetes and fasting blood sugar, lacking social support, relying on oral medication, perceiving limited benefits from self-care practices, and not being a member of diabetic associations were identified as independent factors of poor self-care.

## Introduction

Diabetes mellitus (DM) is a metabolic disorder of multiple etiologies characterized by chronic hyperglycemia with disturbance of carbohydrate, fat, and protein metabolism. It results from a defect in insulin secretion, defective insulin action, or both, which leads over time to serious damage to different organs^1^. According to the International Diabetics Federation (IDF) Diabetes Atlas 2019, there are an estimated 463 million (9.3%) people with diabetes aged 18–79, and the number of people to rise beyond 700 million in less than 25 years worldwide if not controlled. Of these people, 19.4 million adults live in Africa and Ethiopia is one of the members of IDF with 1.7 million people with diabetes^2,3^. Diabetes is a complex chronic illness that needs continuous medical care, in which one's self-care practice plays a significant role in the reduction of life-threatening conditions^4,5^.

Diabetes was known to be a disease in developed countries with rich people. Nevertheless, nowadays it has become a burden globally and specifically in developing countries^6,7^. Increased mortality and morbidity in developing and developed countries are caused by this incurable disease, and the lion's share is type 2 diabetes mellitus (T2DM). The IDF reported that in 2017, 4 million deaths occurred worldwide^8,9^, in contrast significantly higher than the World Health Organization (WHO) (1.3 million) deaths^10^. Diabetes is the 11th leading cause of infirmity worldwide; it is the major cause of new cases of blindness in adults; and it is also the leading cause of end-stage renal disease, accounting for about 20–40% of new cases. More than half of lower limb amputations occur among people with diabetes^11,12^. The economic burden of diabetes is significant across the globe, following the high proportion of deaths < 60 years, which is the workforce at 46.2% worldwide and the highest being Africa with 73.1%^6,13,14^.

Self-care is gradually acquiring skills and developing an understanding of the ways of living with diabetes^15^. Since the majority (95%) of self-care is done by the patient and their families, the patient's ability to practice self-care is the main component in keeping the disease under control and altering the outcomes. These practices include self-monitoring of blood glucose, nutrition, physical activity, monitoring of diabetic complications, and medication adherence^15,16^.

A lack of skills and appropriate knowledge of self-care practices worsens the burden of the disease each year^15,17^. It is confirmed that in lowering admissions and hospital visits, self-care has marked significance^18^. Diabetic self-care practices in Ethiopia range from 23.2 to 62.7%^19,20^. Evidence suggests self-care practice is a preferable approach with cost-effective and restorative results in countries like Ethiopia, where there are scant resources and ever-growing medical costs^17,21^.

As far as the researchers’ knowledge, the level of poor self-care practices and associated factors among people living with diabetes in Adama are not well understood. The present study involved both hospitals and primary healthcare facilities, but most prior studies were conducted in one of these settings. Moreover, factors associated with self-care practice were not well identified in Adama, Ethiopia. Therefore, the researcher aimed to assess the magnitude and associated factors of poor self-care practices in the study setting. The study will contribute to bridging the information gap and is expected to generate updated information.

## Methods and materials

### Study design, period, and population

An institutional-based cross-sectional study design was employed from April 01 to May 30, 2022, in Adama town, located 95 km from Addis Ababa. Adama town is one of Ethiopia's major cities, boasting a total population of 448,462, with 222,355 being male and 226,106 females. The town's healthcare infrastructure consists of one governmental hospital and eight health centers dedicated to providing follow-up care for diabetic patients. The hospital, situated within the town, offers preventive and curative services to over five million people. Additionally, the health centers collectively serve a catchment population of 410,646^22^.

The source population included all patients with T2DM who were receiving follow-up care at public health facilities in Adama town. The study population consisted of randomly selected T2DM patients who were undergoing diabetic follow-up at selected public health facilities in Adama town and had attended care during the study period. All type 2 adult diabetic patients (18 years and above) who had regular follow-ups for at least six months were included in the study. Adults with T2DM who were critically and mentally ill and pregnant women patients diagnosed during the study period were excluded.

### Sampling size determination and sampling procedure

An independent sample size was calculated for the two specific objectives sought in the current study and the largest sample was taken to address both of the study’s aims. The largest sample size was the one calculated using the single population proportion formula which is used to estimate the magnitude of poor self-care practice among T2DM patients. The statistical assumptions for the calculation of sample size such as; a 95% desired level of confidence (95% CI) and a 5% margin of error were considered. Furthermore, the proportion of poor self-care practice (*P*) was considered to be 52% which was obtained from a similar study conducted in Addis Ababa, Ethiopia^23^. Accordingly, the calculated sample size becomes 384. Then, five percent of the calculated sample size was added to compensate for non-response. Consequently, the total sample size of 404 was considered to be incorporated in the current study.

Eight public health facilities in the town were stratified into hospitals and health centers. Using a simple random sampling technique, one hospital and two health centers were selected. The study participants were drawn from 683 patients in the hospital and 559 in the two health centers. Samples were allocated proportionally to each of the selected health facilities based on the expected number of patients attending care during the data collection period. A systematic random sampling technique was used to select study participants independently from each selected facility. The sampling interval at each facility 'k_i_' was determined by dividing the total number of attendants in one month (N_i_) by the required sample size (n_i_) from each selected health facility i.e. k_i_ = N_i_ /n_i_. Patients were selected every three intervals until the total sample size was reached. The random start among the first three attendants was determined using the lottery method.

### Data collection tool

Data were collected using an interviewer-administered structured questionnaire. The questionnaire contains information about socio-demographic factors (such as age, sex, educational status, marital status, occupation, income, residence, and religion), personal factors (including knowledge of diabetes, having a glucometer at home, membership in a diabetic association, social support, and diabetic counseling), clinical factors (such as having comorbidity, duration of diabetes, type of treatment, knowledge on of fasting blood sugar levels, and family history), diabetic health belief (covering perceived susceptibility to DM complications, perceived severity of DM, perceived benefits of self-care practices, and perceived barriers to self-care practices), treatment satisfaction (including the level of satisfaction with treatment and care) and Summary of Diabetes Self-Care Activities (SDSCA) measure.

The self-care practice was measured by the SDSCA questionnaire, which was adopted from a validated SDSCA measure revised from seven studies' results^24^. This tool is commonly employed to evaluate various domains of diabetic self-care practices, including general and specific diet, exercise, medication adherence, self-monitoring of blood glucose (SMBG), and foot care. It consists of 17 items with response choices ranging from 0 to 7, indicating the frequency of self-care activities performed in the last 7 days for each domain. Participants in the study who scored below the mean on the SDSCA were classified as having poor self-care practices, while those who scored at or above the mean were considered to have good self-care practices^17,25^.

The diabetic knowledge section comprises questions adapted from previously validated tools, specifically the revised brief diabetes knowledge test (DKT2)^26^. The DKT2 includes 14 general knowledge items suitable for adults with Type 1 and Type 2 diabetes, along with 9 items related to insulin use appropriate for adults with Type 1 diabetes. For our study, we focused solely on the general knowledge items. For our study, we focused solely on the general knowledge items. Due to their lack of relevance in the Ethiopian context and because they are not recommended for non-US patients, two items were omitted in the present study. Therefore, knowledge was measured using 12 general knowledge items, and each participant's score was calculated by dividing the number of correct answers by the total number of questions. Participants who scored at or above the overall mean value were considered to have adequate knowledge^27^.

The diabetic treatment satisfaction questionnaire (DTSQ) consisted of six items assessing treatment satisfaction, each was scored on a scale of 1–5, with five representing the greatest satisfaction^28^. A five-point Likert scale (1) very unsatisfied, (2) unsatisfied, (3) neutral, (4) satisfied, and (5) very satisfied) was used to grade the level of satisfaction of patients. The diabetic health belief was assessed by adapting 16 item questionnaire, as developed by Given^29^, on perceived susceptibility, perceived severity, perceived benefits, and perceived barriers, to measure the beliefs of diabetic patients about their diabetes which had proven to be reliable in a similar study in Nigeria^30^. Agreement with each item on diabetic health belief was indicated on a 5-point Likert scale ranging from 1 (strongly disagree) to 5 (strongly agree).

The questionnaire was first translated from English to local languages (*Amharic and Afaan Oromoo*) and then translated back to English to ensure consistency and accuracy. Data collection was conducted by four trained BSc nurses from different facilities, alongside two supervisors. A pre-test involving 5% of the total sample of patients at non-selected public health institutions was carried out to ensure the quality and compliance of the data abstraction format and questionnaire with the study’s objectives., and a correction was made accordingly. Data collectors and supervisors were given two days of training on data collection procedures and the goal of the study. All collected data were validated for completeness and consistency during data management, storage, and analysis.

### Data processing and analysis

Data were coded and entered into a computer using Epi-Info Version 7.2 and exported to SPSS version 25 statistical software for processing and analysis. Before analysis data cleaning, coding, and categorizing were performed. Before choosing the appropriate numerical summary measures for continuous variables, normality was checked using the Shapiro–Wilk test. Descriptive statistics were used to summarize and explore the characteristics of patients. The level of poor self-care practices was estimated using proportion along with 95% CI.

The associations between independent variables and self-care practices were modeled using binary logistic regression analysis. The statistical assumptions for binary logistic regression (adequacy of sample in each cross-tabulated result, expected count in each cell) were assessed before fitting the regression model. First simple logistic regression analysis was used to select variables that had a crude association with self-care practice. At this level, the candidate independent variables for multiple regression analysis were selected at *P*-value < 0.25. Multiple logistic regression was applied to identify independent variables significantly associated with self-care practices after adjusting for the effects of possible confounding variables.

The regression model was fitted using a standard model-building approach. The procedure of fitting the model started with subjecting all selected variables to multiple regression, and in the process variables that did not have a significant association with poor self-care practice at a *p*-value < 0.05 were excluded from the model one by one starting with variables with the worst *P*-value. The odds of having poor self-care practice were estimated using an adjusted odds ratio (AOR) with 95% CI. The significance of associations was declared for variables with a *p*-value less than 0.05. The final fitted model was assessed for multicollinearity using the Variance Inflation Factor (VIF) of 10 and goodness of fit using the Hosmer and Lemeshow test.

### Ethics approval and consent to participate

Ethical approval was obtained from Adama Hospital Medical College Institutional Review Board (IRB) with Reference number 0911/k373/14 and permission to conduct the study was secured from respective health facilities. The study was employed following the ethical principles of the Declaration of Helsinki. All participants gave informed consent and confidentiality was maintained by using codes without personal identifiers.

## Results

### Socio-demographic characteristics

In this study, a total of 387 diabetic patients on follow-up care were included giving a response rate of 95.8%. The median (± Interquartile range) age of study participants was 58 (48–65) years. Among diabetic patients, 198 (51.2%) were females and 299 (77.3%) were married (Table 1).

**Table 1:** Socio-demographic characteristics of adults with type 2 diabetes in Adama, Ethiopia, 2022 (n = 387).

Variables	Frequency	Percentage
*Sex*		
Male	189	48.8
Female	198	51.2
*Age in years*		
18–39	31	8.0
40–49	79	20.4
50–59	102	26.4
60 and above	175	45.2
*Place of residence*		
Urban	359	92.8
Rural	28	7.2
*Marital status*		
Single	15	3.9
Married	299	77.3
Divorced	25	6.5
Widowed	48	12.4
*Religion*		
Orthodox	248	64.1
Muslim	73	18.9
Protestant	66	17.1
*Educational status*		
Unable to read and write	68	17.6
Read and write	64	16.5
Primary	81	20.9
Secondary	76	19.6
Diploma	39	10.1
Degree and above	59	15.2
*Occupation*		
Housewife	112	28.9
Farmer	21	5.4
Governmental	85	22.0
Private employee	71	18.3
Merchant	60	15.5
Others**	49	12.3

*ETB* Ethiopian birr, **student, pensioner, No occupation.

One participant may have more than one occupation.

### Clinical characteristics

Among participants, 120 (31%) had a family history of diabetes, 142 (36.7%) had diabetic-related complications, of which 112 (78.9%) had hypertension. Regarding the type of medications, 272 (70.3%) were taking oral medication. Among participating patients, 240 (62.0%) were aware of their FBS status of which 200 (83.3%) of them had FBS levels of 130 mg/dl and above (Table 2).

**Table 2:** Clinical characteristics of adults with type 2 diabetes in Adama, Ethiopia, 2022 (n = 387).

Variables	Number	Percentage
*Have complications*		
Yes	142	36.7
No	245	63.3
*Family history of DM*		
No	267	69
Yes	120	31
*Types of current treatment*		
Insulin	74	19.1
Oral	272	70.3
Both	41	10.6
*Awareness of recent FBS*		
Yes	240	62
No	147	38
*Recent FBS level (n = 240)*		
< 130	40	16.7
> 130	200	83.3

*FBS* Fasting blood sugar, *DM* Diabetes mellitus.

### Personal factors

The study revealed that 160 (41.3%) patients were diagnosed with DM five years back. Among patients, 203 (52.5%) have been receiving diabetic counseling, and 309 (79.8%) were not a member of the diabetic association. Among the participants, 231 (59.7%) had a lower level of knowledge regarding DM, and 201 (51.9%) had infrequent social support (Table 3).

**Table 3:** Personal factors of adults with type 2 diabetes in Adama, Ethiopia, 2022 (n = 387).

Variables	Frequency	Percentage
*Diabetes duration in years*		
Less than 5	160	41.3
5–10	114	29.5
More than 10	113	29.2
*Receive Diabetic counseling*		
Never	151	39
Yes sometimes	203	52.5
Yes regularly	33	8.5
*Source of diabetic counseling (n = 236)*		
Health professionals	185	78.4
Media	34	14.4
Others**	17	7.2
*Having glucometer*		
Yes	143	37
No	244	63
*Member of diabetic association*		
No	309	79.8
Yes	78	20.2
*Level of knowledge on DM*		
Higher	156	40.3
Lower	231	59.7
*Having social support*		
Infrequently	201	51.9
Frequently	186	48.1
*Level of treatment satisfaction*		
Low	167	43.2
High	220	56.8

**Friends, family, and books.

### Diabetic health belief

The result of this study revealed that 222 (57.4%) and 218 (56.3%) patients responded with unfavorable perceived severity and perceived benefit of self-care practices respectively **(**Table 4**).**

**Table 4:** Diabetic health beliefs of adults with type 2 diabetes in Adama, Ethiopia, 2022 (n = 387).

Variables	Frequency	Percentage
*Perceived susceptibility to DM complication*		
Unfavorable	175	45.2
Favorable	212	54.8
*The perceived barrier to self-care practice*		
Unfavorable	199	51.4
Favorable	188	48.6
*Perceived severity of DM*		
Unfavorable	222	57.4
Favorable	165	42.6
*Perceived benefit of self-care practice*		
Unfavorable	218	56.3
Favorable	169	43.7

*DM* Diabetes mellitus.

### Diabetic self-care practices

This study indicated that the magnitude of poor self-care practices was 54% [95% CI 49.1, 58.6]. In stratified analysis, the magnitude of poor self-care practices was 218 (56.3%) on diet, 191 (49.4%) on physical activity, 158 (40.8) on SMBG, and 195 (50.4%) on foot care among respondents.

### Factors associated with self-care practices

From the simple logistic regression analysis, monthly income, marital status, diabetic counseling, having a family history of diabetes, types of current treatment, having a glucometer, being aware of recent FBS, level of knowledge on diabetes, membership of the diabetic association, having social support, perceived susceptibility of DM complication, the perceived barrier of self-care practice, perceived severity of DM, and perceived benefit of self-care practice were selected as a candidate variables for multiple regression at a *p*-value of < 0.25. After adjusting for possible confounders, marital status, types of current treatment, being aware of recent FBS, level of knowledge on diabetes, being a member of the diabetic association, having social support, and perceived benefit of self-care practices showed statistically significant association with poor self-care practices.

Accordingly, divorced patients had 3.5 higher odds of poor self-care practices compared to widowed patients (AOR = 3.5; 95% CI 1.05, 12.25). It was also identified that patients who were on insulin injections alone had 6.3 times (AOR = 6.3; 95% CI 1.98, 20.64) and those who were on oral medications had 8.6 times (AOR = 8.6; 95% CI 3.03, 24.5) higher odds of poor self-care practices compared to those who were taking both insulin injection and oral medications. Furthermore, 2.9 times higher odds of poor self-care practices were documented among diabetic patients who were unaware of their recent FBS compared to those who were aware of the current FBS (AOR = 2.9; 95% CI 1.68, 5.2). Diabetic patients who had a lower level of knowledge of DM had 70% (AOR = 1.70; 95% CI 1.01, 2.86) increased odds of poor self-care practices compared to those with a higher level of knowledge.

Moreover, patients who were not members of the diabetic association had 3.6 (AOR = 3.6; 95% CI 1.70, 7.59) higher odds of poor self-care practices compared to those who were members of the association. The odds of having poor self-care practice among patients having infrequent social support were 2.9 times (AOR = 2.9; 95% CI 1.78, 4.94) higher compared to those who have frequent social support. The odds of having poor self-care practices among patients who had an unfavorable perceived benefit of self-care practices were 84% higher compared to those who had a favorable perceived benefit of self-care practices (AOR = 1.84; 95% CI 1.04, 3.24) **(**Table 5**).**

**Table 5:** Factors associated with self-care practice among adults with type 2 diabetes in Adama, Ethiopia, 2022 (n = 387).

Variable	Self-care practice		COR (95% CI)	AOR (95% CI)
	Poor	Good		
*Marital status*				
Single	4 (26.7)	11 (73.3)	0.5 (0.154, 2.001)	0.24 (0.053, 1.079)
Married	169 (56.5)	130 (43.5)	1.9 (1.065, 3.696) **	1.38 (0.641, 2.982)
Divorced	17 (68.0)	8 (32.0)	3.2 (1.169, 8.996) *	3.5 (1.054, 12.255)*
widowed	19 (39.6)	29 (60.4)	Ref	Ref
*Type of current treatment*				
Insulin injection	29 (39.2)	45 (60.8)	3.1 (1.225, 7.996) *	6.3 (1.983, 20.648) **
Oral medication	173 (63.6)	99 (36.4)	8.4 (3.627, 19.861) ***	8.6 (3.036, 24.507) **
Both	7 (17.1)	34 (82.9)	Ref	Ref
*Awareness of recent FBS*				
Yes	100 (41.7)	140 (58.3)	Ref	Ref
No	109 (74.1)	38 (25.9)	4.0 (2.561, 6.296)***	2.9 (1.683, 5.201) ***
*Knowledge on DM*				
Lower	147 (63.6)	84 (36.4)	2.6 (1.747, 4.030)***	1.70 (1.012, 2.861) *
Higher	62 (39.7)	94 (60.3)	Ref	Ref
*Member of diabetic association*				
No	190 (61.5)	119 (38.5)	4.9 (2.817, 8.728)***	3.6 (1.709, 7.597) ***
Yes	19 (24.4)	59 (75.6)	Ref	Ref
*Having social support*				
Infrequently	139 (69.2)	62 (30.8)	3.7 (2.438, 5.662)***	2.9 (1.781, 4.944) *
Frequently	70 (37.6)	116 (62.4)	Ref	Ref
*Perceived benefit*				
Unfavorable	142 (65.1)	76 (34.9)	2.8 (1.877, 4.309)***	1.84 (1.045, 3.248) *
Favorable	67 (39.6)	102 (60.4)	Ref	Ref

*P < 0.05; **P < 0.01; ***P < 0.00.

## Discussion

This study aimed to assess the level of poor self-care practices and contributing factors among adults with type 2 diabetes in Adama, Ethiopia. The study revealed an overall level of poor self-care practice of 54% [95% CI 49.1, 58.6]. The finding is consistent with studies conducted in Addis Ababa (52%)^23^, Gondar (51.86%)^31^, and Tigray (53.3%)^3^, but it is higher than the study done in the UAE (15.3%)^16^, Iran (26.2%)^32^, and other studies in Ethiopia (Dilla (23.2%)^20^, West Oromia (36.4%)^21^, Diredawa (44.1%)^27^, and Nekemte (45%)^14^. On the other hand, it is lower than the studies' findings in Bahirdar (71.6%)^33^ and Mekelle (62.7%)^19^. The inconsistencies might be due to differences in the cultural and socioeconomic characteristics of the Ethiopian community, as well as the diverse lifestyles within Ethiopia. Additionally, improvements in the healthcare systems over time may have contributed to these inconsistencies. Prior studies assessed self-care practices only over the previous three days, which might have made it difficult to accurately analyze patients' self-care behaviors. In contrast, the current study assessed self-care practices over the last seven days, providing a longer period to capture the frequency of self-care activities. Furthermore, this study measured the number of days individuals practiced self-care, whereas previous studies used simple 'Yes' or 'No' questions to assess self-care practices^3,27,31^. Furthermore, because of the large number of patients in the facilities, waiting times may deter patients from getting the information they require about self-care techniques.

This study revealed statistically significant positive associations between patients who were divorced and poor self-care practices. This result is consistent with studies conducted in Felegehiwot^34^ and Gondar^35^, but contrasts with findings from Addis Ababa^36^ and West Oromia^21^, where married patients were associated with poor self-care practices. The possible reason could be that divorced patients may lack emotional support from loved ones, making it more difficult for them to cope with various problems and focus on diabetic self-care practices^33^.

According to this study, a lower level of knowledge about diabetes and its complications showed increased odds of poor self-care practices. The finding is in line with a study done in western Ethiopia^17^. This can be explained by the fact that less knowledgeable patients may not be conscious of the benefits of self-care practices and prevention regarding the long-term complications of DM ^27,37^. One argument is that having the correct knowledge about DM and self-care practices promotes clarity and minimizes confusion regarding the practice and the medical condition^27^.

In this study, patients who were on oral medication had higher odds of poor diabetes self-care practices as compared to patients who were on insulin and oral medications. Furthermore, patients who were taking only insulin injections had higher odds of poor self-care practices as compared to those who took both insulin and oral medication. The associations between types of medications and diabetic self-care practices are inconsistent. A study in Iran indicated insulin injection was significantly associated with good self-care practice^32^, while a study done in Bahir Dar town in Ethiopia reported contrasting results^33^. One reason could be that these individuals may have DM that is not controlled by monotherapy (tablets or insulin alone). Moreover, patients who take a single medication may have had diabetes for a shorter duration than those who are on both insulin and oral therapies. Therefore, they might be more concerned about medication side effects than the long-term complications of DM due to their limited knowledge and experience compared to those with a longer duration of diabetes^38,39^.

This study showed that DM patients who did not know their fasting glucose level had greater odds of poor self-care practices as compared to their counterparts. This is in line with a study conducted in Tigray, Ethiopia^19^. Potential explanations include the possibility that individuals unaware of their blood glucose level may not feel motivated to engage in necessary self-care activities, such as eating a healthy diet, exercising, reducing risk, and following recommended self-care guidelines^19,40^.

In this study, the absence of membership in the diabetic association was another important variable statistically associated with poor self-care practices. The finding is in agreement with a study in Ethiopia, which reported higher odds of poor self-care practices among DM patients who were not members of DM associations^34^. The lack of regular monthly diabetic education and support provided to patients, such as access to medication for some lower-income members and relatively inexpensive blood glucose testing, could explain this association. Additionally, patients who are not members of the diabetic association may miss out on receiving support and exchanging beneficial experiences^41^.

The lack of social support was found to be significantly associated with poor self-care practices. Similar findings were observed in studies conducted in Anand Gujarat India^4^, Addis Ababa^23^, and West Shoa Oromia^42^. Social support, whether in the form of emotional, financial, or informational assistance from family or friends, can provide patients with the psychological resilience needed to cope with the complexities of managing diabetes. There is a pressing need for a more supportive environment, as effective social support may facilitate behavioral adjustments leading to improved self-care practices^3,27,31,43^. Therefore, DM patients who lack adequate social support may struggle to implement essential changes in their physical and dietary habits.

Finally, an unfavorable attitude towards the perceived benefits of self-care practices was significantly associated with poor self-care practices. This finding aligns with studies conducted in Northern Ethiopia^19^ and Nigeria^30^. This is sensible because a negative attitude towards self-care weakens beliefs about the expected benefits and healthy behaviors^44^. Moreover, these findings could be attributed to a lack of formal diabetes education, which can influence attitudes toward diabetic self-care behaviors^19,45,46^.

### Strengths and limitations of the study

This study's strength lies in its collection of primary data from multiple care centers, providing a more representative sample. However, it has certain limitations. Firstly, being a cross-sectional study, it cannot demonstrate causal relationships between variables. Secondly, Second, because the study asks about patients' self-care activities during the past seven days and is based on self-reports, the performance of their behaviors was not observed and could not be confirmed. Additionally, since data was collected through interviewer-administered methods by healthcare providers, responses may be influenced by social desirability biases.

## Conclusion

The study revealed that a significant proportion of type 2 diabetes patients had poor diabetes self-care practices, although these practices are essential for controlling diabetes and preventing its complications. Being divorced, having a low level of knowledge about diabetes and fasting blood sugar, lacking social support, relying on oral medication, perceiving limited benefits from self-care practices, and not being a member of diabetic associations were identified as independent factors contributing to poor self-care.

### Recommendations

Healthcare providers should focus on patients who exhibit the aforementioned characteristics. Plans should also be developed to support diabetic individuals in practicing greater self-care. The Ethiopian Diabetes Association needs to advocate for the benefits of membership as well as advise and empower patients to adhere to the recommended diabetes self-care practices. It is recommended that patients join nearby diabetic associations to be able to receive support, affection, and help. Family members should be informed about their important roles in encouraging patients to adopt recommended self-care practices. Future studies should investigate barriers to self-care practices using a qualitative study.

## Data Availability

The datasets generated and/or analyzed during the current study are not publicly available due to participant data protection regulations but are available from the corresponding author upon reasonable request.

## Abbreviations

AOR: Adjusted odds ratio
COR: Crudes odds ratio
DKT: Diabetic knowledge test
DM: Diabetes mellitus
DTSQ: Diabetic Treatment Satisfaction Questionnaire
FBS: Fasting blood sugar
IDF: International Diabetic Federation
SDSCA: Summary of self-care activities
SMBG: Self-monitoring of blood glucose
SPSS: Statistical package for social science
T2DM: Type 2 diabetes mellitus
